# Foliar Spraying with Compound Amino Acid-Iron Fertilizer Increases Leaf Fresh Weight, Photosynthesis, and Fe-S Cluster Gene Expression in Peach (*Prunus persica* (L.) Batsch)

**DOI:** 10.1155/2020/2854795

**Published:** 2020-05-25

**Authors:** Yuting Sheng, Hao Cheng, Limin Wang, Jingyuan Shen, Meiling Tang, Meixia Liang, Kai Zhang, Hongxia Zhang, Qun Kong, Mingliang Yu, Zhizhong Song

**Affiliations:** ^1^College of Agriculture, Ludong University, Yantai 264025, China; ^2^Key Laboratory of Molecular Module-Based Breeding of High Yield and Abiotic Resistant Plants in Universities of Shandong (Ludong University), Yantai 264025, China; ^3^Ministry of Education Key Laboratory for Biodiversity and Ecological Engineering, Institute of Biodiversity Science, Fudan University, Shanghai 200433, China; ^4^Hainan Key Laboratory for Biosafety Monitoring and Molecular Breeding in Off-Season Reproduction Regions, Haikou 570100, China; ^5^Yantai Academy of Agricultural Sciences, Yantai 264000, China; ^6^School of Economics and Management, Northeast Agricultural University, Harbin 150000, China; ^7^Institute of Pomology, Jiangsu Academy of Agricultural Sciences, Nanjing 210014, China

## Abstract

As one of the most important micronutrients, iron (Fe) plays a critical role in various metabolic processes during plant growth and development. However, the molecular mechanisms towards Fe metabolism and nutrition in fruit trees are largely unknown. In this study, we examined the effects of amino acid-Fe compound fertilizer spraying on leaf development in peach (*Prunus persica* (L.) Batsch) at different developmental stages. Foliar spraying with amino acid-Fe compound fertilizer did not cause any significant changes in leaf morphology but remarkably increased leaf fresh weights. Fe concentration, photosynthetic parameter, and Fe-S protein analyses revealed that Fe accumulation, total chlorophyll content, net photosynthetic rate (*P*_N_), and stomatal conductance (*g*_s_), as well as nitrite reductase (NIR) and succinate dehydrogenase (SDH) activities, were significantly higher in the leaves sprayed with amino acid-Fe compound fertilizer than in the control leaves sprayed with distilled water. Further quantitative real-time PCR (qRT-PCR) analyses demonstrated that Fe-S cluster biosynthesis genes were differentially expressed in the leaves at different developmental stages. Foliar spraying with amino acid-Fe compound fertilizer significantly increased the expression of the most tested Fe-S cluster biosynthesis genes. Our findings provide new insights into the understanding of effects of Fe fertilization application on leaf development in perennial woody fruit trees.

## 1. Introduction

Fe deficiency is one of the major micronutrient factors affecting the growth and development of plants, especially fruit trees grown in calcareous and/or alkaline soils, on which fruit yield and quality are dominantly dependent on the growth of one-year-old branches [1–7]. Previous studies have demonstrated that foliar spraying with different compound Fe preparations or fertilization in soil enhanced fruit yield and improved fruit quality in pear [2], grape [3], kiwifruit [8, 9], pear-jujube [10], and nectarine [11]. However, all these studies were mainly focused on biochemical and physiological analyses. The molecular mechanisms towards Fe nutrition and Fe metabolism in fruit trees are still largely unclear.

In higher plants, the metabolism and usage of Fe nutrient are mainly embodied in iron-sulfur (Fe-S) proteins which are involved in many critical metabolic pathways such as photosynthesis, respiration, and DNA repair [12–16]. As typical Fe-S proteins, nitrite reductase (NIR) functions in chloroplastic nitrogen assimilation, and succinate dehydrogenase (SDH) and aconitase (ACO) play crucial roles in the mitochondrial citric acid cycle of glycometabolism [12]. A typical Fe-S cluster biosynthesis process contains an iron donor, a sulfur donor (like NFS), some scaffold (SUFB, SUFC, SUFD, NFU, etc.), and delivery proteins (ISA, GRX, HSCA, etc.), which have been identified in the plastid, mitochondria, cytosol, and nucleus (reviewed in [15, 16]). In *Arabidopsis*, the plastid SUF (the sulfur mobilization) and mitochondrial ISC (iron-sulfur cluster) machineries were independent, whereas the cytosolic CIA (cytosolic iron-sulfur cluster) machinery was dependent on mitochondrial ISC Fe-S assembly [15, 17]. To date, over forty Fe-S cluster biosynthesis genes have been identified in *Arabidopsis* [15, 16], rice [18], soybean [19], and peach [20].

The genomic sequencing of Peach (*Prunus persica* L. Batsch), one of the most popular fruit trees, has been finished [21]. “Xiacui” is an early-mid ripening and nonmelting flesh peach cultivar that ripens in early July when grown in Nanjing area of China [22]. However, both the available soil Fe concentration (0.17 g kg^−1^) and the Fe activation rate (0.54%) in the surface soil in Nanjing area were relatively low and far less than the other trace elements [23, 24]. Previously, we investigated the physiological and transcriptional responses to abiotic stress in the Fe-S cluster assembly pathway in peach seedlings [25] and the expression patterns of Fe-S cluster biosynthesis genes during peach flowering [26] and fruit development [27]. In this work, we analyzed the effects of compound amino acid-Fe fertilizer spraying on leaf growth, Fe accumulation, and Fe-S cluster biosynthesis gene expression in the leaves at different developmental stages in seven-year-old peach trees.

## 2. Materials and Methods

### 2.1. Plant Material and Growth Conditions

Seven-year-old “Xiacui” peach trees grown in the experimental orchard of the National Peach Germplasm Repository (Nanjing, China) were used. Peach trees at the same size grown under the same field condition were divided into two plots, each containing eighteen peach trees to be treated for three biological replicates (six trees each). Each tree in the experiment plot was sprayed with 2 liters of compound amino acid-Fe fertilizer, with a molar ratio of amino acetic acid to Fe_2_SO_4_·7H_2_O at 0.6 : 1, at three leaf developmental stages (March 15th, 2018, leaf bud breaking stage; April 11th, 2018, leaf expanding stage; and July 11th, 2018, leaf full size stage). The final concentration of Fe_2_SO_4_ was 1000 mg kg^−1^, and the pH value was adjusted to 4.5 using ammonium hydroxide. Trees in the control plot were sprayed with 2 liters of distilled water instead. To check the effects of fertilizer spraying on leaf development, the fifth leaves from the apex of one-year-old fruiting shoots were collected on April 10th (leaf expanding stage), July 10th (leaf full size stage), October 15th (defoliation stage), and November 15th (defoliation ending stage), 2018.

### 2.2. Physiological Analyses

At least one hundred leaves were collected randomly for each replicate and then weighed to obtain the total fresh weight at each sampling time. Leaf vertical length and transverse length were calculated by measuring 40 out of 100 leaf samples. For photosynthetic analyses, net photosynthetic rate (*P*_N_), stomatal conductance (*g*_s_), and transpiration rate (*T*_r_) at the second leaf from the apex on one-year-old fruiting shoots were examined with a portable photosynthetic system LI-6400 (LI-COR, Lincoln, Nebraska, USA) [25]. For leaf total chlorophyll assays, leave samples were cut into small pieces and gently ground in a motor and pestle, soaked in 95% ethanol at 4°C in darkness for 12 h, and then centrifuged at 1,000 *g*, at 4°C for 10 min [25]. The supernatant was used to determine absorbance chlorophyll *a* at 665 nm and chlorophyll *b* at 649 nm on a Bio-Rad SmartSpec 3000 spectrophotometer (Wadsworth, Illinois, USA) and then obtain the total chlorophyll content. For Fe concentration analyses, dried leaf samples were digested using the HNO_3_-HClO_4_ method and measured on ICP-AES systems (IRIS Advantage, Thermo Electron, Waltham, USA). ACO, NIR, and SDH activities were determined using NIR, SDH, and ACO Detection Kits (Nanjing Jiancheng Bioengineering Institute, Nanjing, China), following the manufacturer's instruction. One unit of ACO activity was defined as the enzyme amount that isomerized 1.0 *μ*mol of citrate to isocitrate per min at pH 7.4. One unit of NIR activity was defined as the enzyme amount required to catalyze the reduction of 1 *μ*mol NO^2-^, and one unit of SDH activity was defined as the enzyme amount required to decrease the rate of FAD reduction by an absorbance change of 0.01 per min in absorbance recorded at 600 nm. Three independent sampling replicates obtained from the six trees were carried out in each experiment during the same year.

### 2.3. RNA Extraction and Quantitative Real-Time PCR

Total RNAs were extracted from the leaf samples using a MiniBEST Plant RNA Extraction Kit (TaKaRa, Dalian, China) and then were reverse transcribed into cDNA using a PrimeScript™ RT Reagent Kit (TaKaRa, Kyoto, Japan). qRT-PCR was carried out on a 7500 Real-Time PCR System (Applied Biosystems, New York, USA), using a SYBR Premix Ex Taq reaction kit (TaKaRa, Kyoto, Japan). The peach *Ubiquitin* (GenBank No. KJ598788) and *Actin* (KP690196) genes were used as internal controls [20, 28]. No significant difference was observed in the relative expression level of each gene after normalized to both *Ubiquitin* and *Actin*. Therefore, data normalized to *Ubiquitin* were used in this study. Primer sequences of Fe-S cluster biosynthesis and reference genes were listed in Supplementary Table 1. qRT-PCR reactions were carried out as follows: 95°C for 30 sec, 40 cycles of 95°C for 5 sec and 60°C for 34 s, and then 72°C for 60 sec. To calculate the primer efficiency, melting curve, starting template concentration, and PCR efficiency for each sample, the linear regression of the log (fluorescence) per cycle number data was used by taking the logarithm on both sides of an equation as follows: log (*Nc*) = log (*No*) + log (*Eff*) × *C*, where *Nc* is the fluorescence, *No* is the initial concentration of a transcript, *Eff* is the efficiency, and *C* is the cycle number [29]. Relative expression levels of the target genes were presented after normalization to the internal control from three independent biological replicates. The relative expressions were calculated using a log2 scale, and the heatmap was plotted using HemI software according to the method described by Deng et al. [30].

### 2.4. Statistical Analysis

Data were statistically analyzed using Student's *t*-test in the SPSS 13.0 software (SPSS, Chicago, IL, USA). Data were compared between the control and treatment leaves. Correlation analysis was carried out using Pearson correlation in the SPSS 13.0 software (SPSS, Chicago, IL, USA).

## 3. Results

### 3.1. Foliar Spraying with Compound Amino Acid-Fe Fertilizer Enhances Leaf Fresh Weight and Photosynthesis

**Figure 1:**
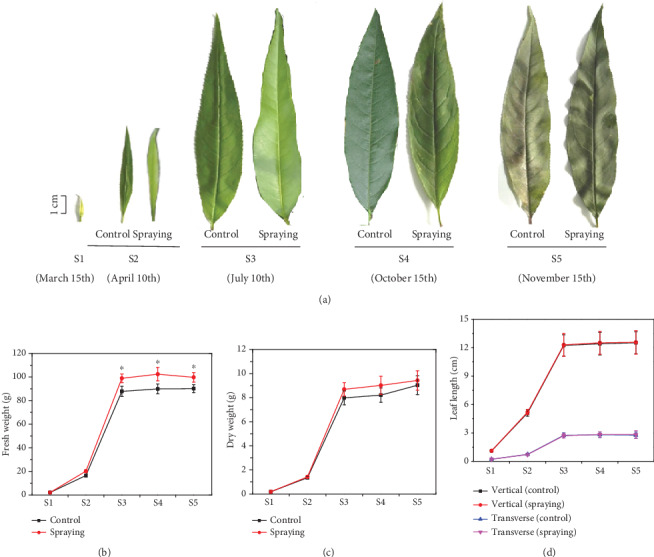
Effects of foliar compound amino acid-Fe fertilizer spraying on leaf development in “Xiacui” peach trees. (a) Photos to show the phenotype leaves at different developmental stages sprayed with distilled water (control) and compound amino acid-Fe fertilizer. (b, c) Fresh and dry weights of leaves in (a) were statistically analyzed. (d) Leaf vertical length and transverse length assays. Leaf samples were collected on March 15th (S1), before the spraying treatment, April 10th (S2), July 10th (S3), October 15th (S4), and November 15th (S5), in 2018. Each tree was sprayed with two liters of compound fertilizer on March 15th, April 11th, and July 11th, respectively, or 2 liters of distilled water (control). The fifth leaf from the apex on one-year-old fruiting branches was collected on April 10th (S2), July 10th (S3), and October 15th (S4), respectively. Data are the means of values (*n* = 3) obtained from three independent sampling replicates ± SE.

To understand the possible effects of compound amino acid-Fe fertilizer spraying on the growth of leaves, leaf morphologies at different developmental stages were observed during the years 2016-2018. Based on the *Descriptors and Data Standard for Peach* [31], leaf development was divided into five stages. The first stage (S1), embodied on March 15th, is the leaf bud breaking stage. At this stage, about 5% of leaf bud scales were split with apices that appeared at the top of leaf buds, and leaf fresh weight was extremely low due to the small size (Figure 1(a)). The second stage (S2), embodied on April 10th, is the leaf expanding stage. At this stage, about 5% of the first leaves were fully expanded, and leaf fresh weight and chlorophyll content increased significantly (Figure 1(a) and Table 1). The third stage (S3), embodied on July 10th, is the full size leaf stage. At this stage, leaf growth reached its final full size and weight (Figure 1(a)), accompanied with the maximum total chlorophyll content and photosynthesis characteristics, i.e., *P*_N_, *g*_s_, and *T*_r_ (Table 1). Meanwhile, the fruits on branches are ripening and ready to be picked up for the market. The fourth stage (S4), embodied on October 15th, is the defoliation stage. At this stage, about 25% of mature leaves fell off the trees naturally. The last stage (S5), embodied on November 15th, is the defoliation ending stage. At this stage, about 95% of the senescence leaves fell off the trees (Figure 1(a)).

Foliar application of compound amino acid-Fe fertilizer did not cause any morphological change in the leaves, since leaf size and shape were about the same on the trees sprayed with either distilled water (control) or compound amino acid-Fe fertilizer at all developmental stages (Figure 1(a)). However, the fresh weight of leaves sprayed with compound amino acid-Fe fertilizer was significantly higher than that of the control leaves, although no significant difference was observed in leaf dry weight, vertical length, and transverse length (Figures 1(b)–1(d)). We then examined the total chlorophyll content and photosynthesis in both the control and compound amino acid-Fe fertilizer-sprayed leaves. We found that from stage S3 to stage S5, total chlorophyll content, *P*_N_, *g*_s_, and *T*_r_ decreased gradually in both the control and fertilizer-sprayed leaves. However, the total chlorophyll content, net photosynthetic rate, and stomatal conductance in the leaves sprayed with compound amino acid-Fe fertilizer were significantly higher than those in the control leaves at the same developmental stage (Table 1).

### 3.2. Fe Concentration and Fe-S Protein Enzyme Activity Are Increased in the Leaves Sprayed with Compound Amino Acid-Fe Fertilizer

To understand the physiological effects of foliar compound amino acid-Fe fertilizer spraying, we investigated the content of Fe and enzyme activity of Fe-S proteins in the leaves sprayed with distilled water and compound amino acid-Fe fertilizer. Similar to the changes in total chlorophyll content and photosynthesis, Fe concentration was significantly higher in the leaves sprayed with amino acid-Fe fertilizer from stage S2 to stage S5 (Table 2). Leaf Fe concentration increased by 25.80%, 24.65%, and 21.64% at stage S2, stage S3, and stage S4, respectively. Similarly, the enzyme activity of NIR and SDH, two indispensable Fe-S proteins involved in plant metabolism [12], was also remarkably higher. The SDH activity increased by 16.50%, 15.51%, and 21.08% at stage S2, stage S3, and stage S4, respectively. And the NIR activity increased by 19.61% at stage S2 and 21.08% at stage S3 but about the same at stages S4 and S5. Different from NIR and SDH, no significant difference was observed in the ACO activity at all developmental stages (Table 2). The activities of NIR and SDH were positively correlated to leaf Fe concentrations, especially from stage S1 to stage S4.

**Figure 2:**
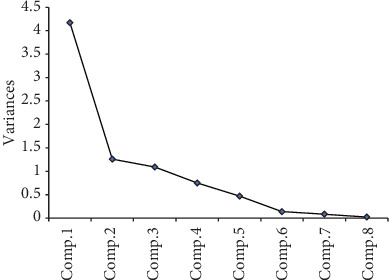
Principal component analysis between treatment and variable interaction. Comp.1, total chlorophyll content; Comp.2, net photosynthetic rate (*P*_N_); Comp.3, stomatal conductance (*g*_s_); Comp.4, transpiration rate (*T*_r_); Comp.5, Fe concentration; Comp.6, nitrite reductase (NIR) activity; Comp.7, succinate dehydrogenase (SDH) activity; Comp.8, aconitase (ACO) activity.

We further performed principal component analysis (PCA). Base on the principle that the characteristic root is greater than or equal to 1, three classes of principal components were extracted. The contribution rates to the effects of foliar fertilizer spraying on leaf development were 52.5%, 16.7%, and 14.6% for the first, second, and third classes, respectively, with a total contribution rate of 83.81% (Figure 2). Among the principal components in the first class, SDH activity, total chlorophyll content, and *P*_N_ exhibited the largest loads. And in the second and third classes, Fe concentration, *g*_s_, and NIR and ACO enzyme activities showed the largest loads (Figure 2). Therefore, SDH activity, total chlorophyll content, and *P*_N_ in the first class were closely associated with the phenotypes observed in response to foliar spraying treatment.

### 3.3. Different Expression Patterns of Fe-S Cluster Biosynthesis Genes during Leaf Development

**Figure 3:**
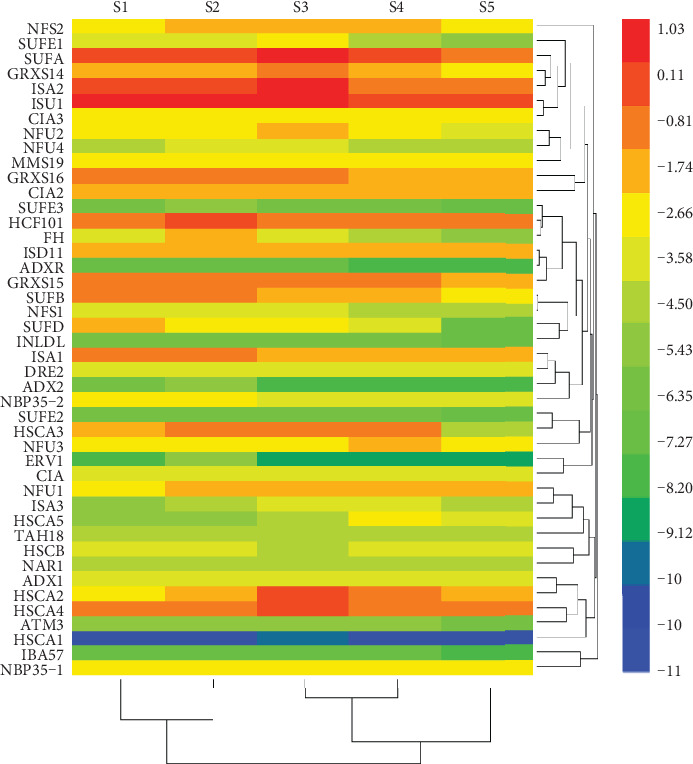
Fe-S cluster biosynthesis genes were differentially expressed at different leaf developmental stages. Leaf samples were collected on March 15th (S1), before the spraying treatment, April 10th (S2), July 10th (S3), October 15th (S4), and November 15th (S5), in 2018. The relative expression level of genes was presented after normalization to the internal control *Ubiquitin* and calculated using a log2 scale. The heatmap was plotted using HemI software according to the method described by Deng et al. [30]. The red and blue boxes indicate the high and low expression levels, respectively.

Fe-S cluster biosynthesis genes play important roles in Fe metabolism. Therefore, we examined the expression patterns of Fe-S cluster biosynthesis genes at different leaf developmental stages by qRT-PCR analyses. As shown in Figure 3, Fe-S cluster biosynthesis genes were differentially expressed in the leaves at different developmental stages. The expression level of *ISU1* was the highest, followed by *ISA2* and *SUFA*, whereas the expression levels of *SUFE3*, *ADX2*, *INDL*, *ATM3*, and *ERV1* were extremely low, and *SUFE2*, *ADXR*, *HSCA1*, and *IBA57* were hardly detected, at all the tested stages. It is noteworthy that the expression of both plastid SUF and mitochondrial ISC machinery genes increased from stage S2 to stage S3, then gradually decreased from stage S4 to stage S5. However, the expression of all cytosolic CIA machinery genes, except *NBP35*-*2* which was highly expressed at stages S1 and S2 and decreased at stages S3 and S5, was pretty stable throughout all the developmental stages (Figure 3).

### 3.4. Promoted Fe-S Cluster Biosynthesis Gene Expressions in the Leaves Sprayed with Compound Amino Acid-Fe Fertilizer

**Figure 4:**
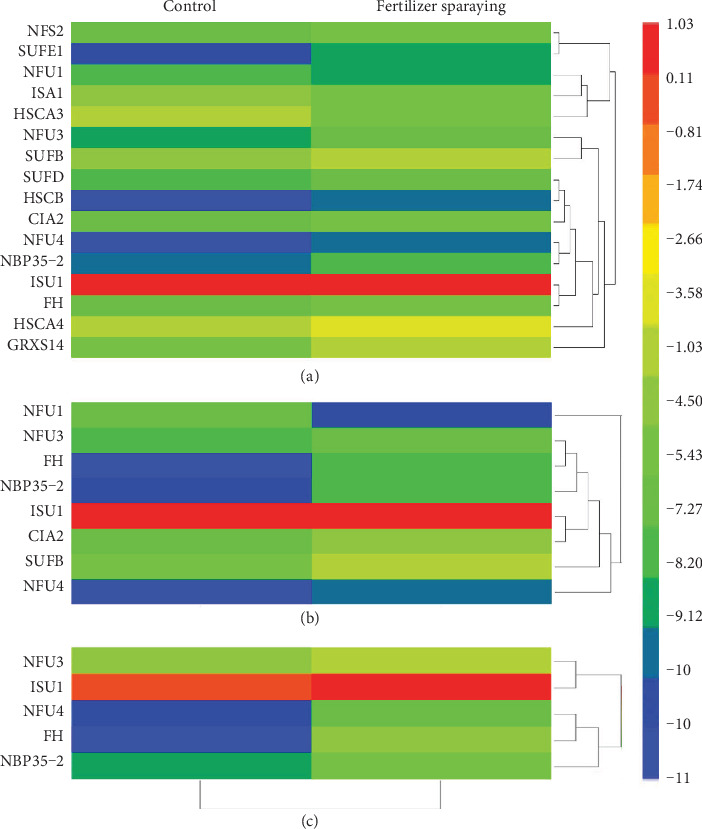
Induced expression of Fe-S cluster biosynthesis genes in the leaves sprayed with compound amino acid-Fe fertilizer. (a) Gene expression in the leaves collected at stage S2 on April 10th, 2018. (b) Gene expression in the leaves collected at stage S3 on July 10th, 2018. (c) Gene expression in the leaves collected at stage S4 on October 15th, 2018. The relative expression level of genes was presented after normalization to the internal control *Ubiquitin* and calculated using a log2 scale. The heatmap was plotted using HemI software according to the method described by Deng et al. [30]. The red and blue boxes indicate the high and low expression levels, respectively.

To further understand the effects of compound amino acid-Fe fertilizer spraying on the expression of Fe-S cluster biosynthesis genes, we compared their expression levels in the leaves sprayed with distilled water and compound amino acid-Fe fertilizer. qRT-PCR analyses indicated that the expression of the most tested Fe-S cluster biosynthesis genes was induced by the fertilizer spraying at stages S2, S3, and S4. Among the sixteen tested Fe-S cluster biosynthesis genes (7 plastid SUF, 7 mitochondrial ISC, and 2 cytosolic CIA) including seven scaffold protein encoding genes (*NFU1*, *NFU3*, *SUFB*, *SUFD*, *ISU1*, *NFU4*, and *NBP35*-*2*), most of them, except *NFU1*, *ISA1*, and *HSCA3*, which were reduced, were induced by fertilizer spraying at stage S2 (Figure 4(a)). At stage S3, expression levels of eight Fe-S cluster biosynthesis genes (3 plastid SUF, 3 mitochondrial ISC, and 2 cytosolic CIA) were significantly increased (Figure 4(b)). At stage S4, expression levels of five Fe-S cluster biosynthesis genes (2 plastid SUF, 2 mitochondrial ISC, and 1 cytosolic CIA) were significantly increased by the fertilizer spraying (Figure 4(c)). Notably, the expression levels of *FH*, an iron donor encoding gene, and 4 scaffold protein encoding genes, *NFU3*, *ISU1*, *NFU4*, and *NBP35*-*2*, were persistently induced at all tested stages (S2, S3, and S4).

## 4. Discussion

As one of the most indispensable mineral elements, Fe favorably affects plant growth and development [1, 2, 5]. The growth and development of peach leaves were not affected by compound amino acid-Fe fertilizer spraying, since no significant difference in the size and shape was observed between the leaves sprayed with distilled water and compound amino acid-Fe fertilizer (Figures 1(a), 1(c), and 1(d)). However, leaf fresh weight was remarkably higher upon the fertilizer spraying, although leaf dry weight was not changed significantly (Figure 1(b)). Therefore, water holding capacity in the mesophyll cells may be promoted upon the fertilizer spraying.

Fe metabolism and usage in plants are mainly mediated by Fe-S proteins that function in various metabolic pathways, including photosynthesis and respiration [12–14, 16]. The increased chlorophyll content and photosynthetic properties (*P*_N_, *g*_s_, and *T*_r_) in the leaves sprayed with compound amino acid-Fe fertilizer implied that Fe was an indispensable micronutrient during leaf development, especially at the leaf expanding and full size mature stages (Table 1). The increased Fe concentration, as well as the augmented NIR, SDH, and ACO enzyme activities, in the leaves sprayed with compound amino acid-Fe fertilizer further revealed the importance of Fe nutrition during leaf development in peach (Table 2). Further correlation analyses demonstrated that leaf Fe nutrition at stage S3 was positively correlated to leaf weight, size, chlorophyll content, *P*_N_, *g*_s_, *T*_r_, and the NIR, SDH, and ACO enzyme activities. Therefore, sufficient Fe may be required at this stage to maintain the normal Fe-dependent metabolisms and processes, including photosynthesis, respiration, and coenzyme synthesis.

Fe-S cluster biosynthesis genes were differentially expressed at different leaf developmental stages (Figure 3). Expression of plastid SUF and mitochondrial ISC machinery genes gradually increased and reached their highest expression levels at the full size leaf stage (S3), whereas expressions of all cytosolic CIA machinery genes were only slightly changed. Therefore, plastid SUF and mitochondrial ISC machinery genes may play a more important role in the leaf expanding and full size formation stages, a key period for the fast-growing and ripening of fruits. Moreover, more than 36% of peach Fe-S cluster biosynthesis genes were induced by fertilizer spraying at stage S2 (Figure 4). We postulated that fertilizer treatment favorably enhanced the transcript levels of Fe-S cluster biosynthesis genes in peach leaves that further strengthened Fe accumulation and facilitated Fe-dependent metabolisms, as indicated by the increased NIR and SDH enzyme activities (Table 2).

The expression patterns of Fe-S cluster biosynthesis genes in the leaves of seven-year-old trees are slightly different from those in the flowers and one-month-old seedlings. In the leaves of seven-year-old trees, *ISU1*, *ISA2*, and *SUFA* were the most abundant genes expressed, whereas in the leaves of one-month-old peach seedlings and in peach flowers, *ISU1*, *GRXS14*, and *ISA1* and *ISU1*, *HSCA4*, and *HSCA2*, respectively, were the most abundant genes expressed [25, 26]. Nonetheless, *ISU1* was the most abundant gene expressed in all tested tissues or organs of peach trees. As a scaffold protein encoding gene [32, 33], expression of *ISU1* increased at all stages upon the fertilizer treatment. We speculated that ISU1 is an essential or dominant scaffold protein for mitochondrial Fe-S cluster biosynthesis in peach trees. Further correlation analysis also demonstrated that *ISU1* gene expression was positively correlated to *P*_N_ and the NIR and SDH activities (Table 3).

The expression of *HSCA1* could be hardly detected, but a moderate expression of the other three homologs, *HSCA2*, *HSCA3*, and *HSCA4*, was detected at all tested stages, which was similar to that in one-month-old peach seedlings [25] and flowers [26]. At stage S2, expression of *HSCA3* decreased, while expression of *HSCA4* increased, with a slight expression change of *HSCA2*, upon the fertilizer spraying treatment (Figure 4(a)). Therefore, *HSCA2*, *HSCA3*, and *HSCA4* may function as major chaperones in the mitochondrial Fe-S cluster biosynthesis pathway in peach, especially after Fe fertilizer application. Moreover, expression of *ADXR* [34, 35] was not detected in the leaves, while the other three electron transfer encoding genes, *ADX1*, *TAH18*, and *DRE2*, were moderately expressed in one-month-old peach seedlings [25], flowers [26], and fruits [27], indicating that peach trees prefer to use ADX1, TAH18, and DRE2 as electron transfers. *IBA57*, an aminomethyltransferase [35, 36], was the lowest gene expressed in the leaves, one-month-old peach seedlings [25], and fruits [27] but was moderately expressed in flowers [26], implying that this gene may have functional roles during peach flowering and fruit development but not leaf development from the long-term evolution. In *Arabidopsis*, *AtSUFE2* was specifically expressed in flowers [37, 38]. Similarly, *SUFE2* was mainly expressed in the pollen of peach flowers [26] but could not be detected in one-month-old peach seedlings [25], fruits [27], and the leaves we tested. These findings further proved that *SUFE2* may have special functions in peach pollen formation or development.

It has been well documented that sufficient functional scaffold proteins are required for plant Fe-S cluster biosynthesis [12, 16]. The increased expression of scaffold protein encoding genes (*ISU1*, *NFU3*, *NFU4*, and *NBP35*-*2*) in the fertilizer-sprayed leaves suggested that scaffold proteins were urgently required for Fe-S cluster biosynthesis. Recently, it was reported that *FH* was involved in the regulation of NFS1 activity in *Arabidopsis* mitochondria [39]. Considering its higher expression at the leaf expanding stage and steady response to fertilizer spraying treatment, we speculated that *FH* is necessary for peach mitochondrial ISC assembly and may be a special signal sensing factor upon fertilizer spraying. Therefore, all Fe-S cluster biosynthesis genes cooperate with each other exquisitely to play crucial functions in maintaining the internal Fe nutritional status in the Fe-dependent metabolic pathways. Our findings provide new insights into the effects of compound amino acid-Fe fertilizer on leaf development in peach.

## 5. Conclusions

The morphological development of leaves on “Xiacui” peach trees was not affected by the foliar spraying with compound amino acid-Fe fertilizer. The increased leaf chlorophyll content, photosynthesis properties (*P*_N_, *g*_s_, and *T*_r_), Fe-S enzyme activities (NIR, SDH, and ACO), and Fe-S cluster biosynthesis gene expression were positively correlated to the augmented leaf Fe concentration after foliar spraying with compound amino acid-Fe fertilizer.

## Figures and Tables

**Table 1:** Effects of foliar fertilizer spraying on leaf chlorophyll content and photosynthetic properties at different leaf developmental stages^a^.

Date	Total chlorophyll content (g·kg^−1^)		Net photosynthetic rate (*μ*mol·m^−2^·s^−1^)		Stomatal conductance (vpm)		Transpiration rate (mmol·m^−2^·s^−1^)	
	Control	Fertilizer	Control	Fertilizer	Control	Fertilizer	Control	Fertilizer
March 15th (S1)	0.88 ± 0.06	—	—	—	—	—	—	—
April 10th (S2)	1.44 ± 0.07	1.69 ± 0.06^∗^	23.57 ± 0.19	26.98 ± 0.27^∗^	0.43 ± 0.03	0.55 ± 0.03^∗^	3.69 ± 0.12	4.22 ± 0.09^∗^
July 10th (S3)	1.56 ± 0.05	1.82 ± 0.07^∗^	29.68 ± 0.23	34.37 ± 0.31^∗^	0.51 ± 0.04	0.64 ± 0.05^∗^	4.03 ± 0.26	4.27 ± 0.17
October 15th (S4)	1.01 ± 0.06	1.22 ± 0.06^∗^	18.82 ± 0.12	22.06 ± 0.39^∗^	0.29 ± 0.02	0.39 ± 0.02^∗^	2.61 ± 0.21	3.05 ± 0.24
November 15th (S5)	0.22 ± 0.03	0.24 ± 0.04	4.25 ± 0.06	4.52 ± 0.24	0.08 ± 0.01	0.10 ± 0.02	1.08 ± 0.13	1.12 ± 0.11

^a^No detection. ∗ stands for *t*-test under *P* < 0.05.

**Table 2:** Effects of foliar fertilizer spraying on Fe concentration and Fe-S proteins involved in plant metabolism at different leaf developmental stages^a^.

Date	Fe concentration (g·kg^−1^ (DW))		Nitrite reductase (U·mg^−1^ (protein))		Succinate dehydrogenase (U·mg^−1^(protein))		Aconitase (U·mg-1(protein))	
	Control	Fertilizer	Control	Fertilizer	Control	Fertilizer	Control	Fertilizer
March 15th (S1)	0.94 ± 0.08	—	2.83 ± 0.11	—	5.21 ± 0.09	—	0.29 ± 0.03	—
April 10th (S2)	1.24 ± 0.07	1.56 ± 0.09^∗∗^	3.57 ± 0.13	4.27 ± 0.12^∗^	6.43 ± 0.04	7.49 ± 0.03^∗^	0.34 ± 0.04	0.35 ± 0.03
July 10th (S3)	1.42 ± 0.10	1.77 ± 0.12^∗∗^	4.36 ± 0.12	4.71 ± 0.14^∗^	6.51 ± 0.09	7.52 ± 0.05^∗^	0.35 ± 0.03	0.37 ± 0.02
October 15th (S4)	1.34 ± 0.13	1.63 ± 0.13^∗^	3.32 ± 0.12	3.56 ± 0.19	3.13 ± 0.05	3.79 ± 0.02^∗∗^	0.29 ± 0.01	0.32 ± 0.05
November 15th (S5)	1.23 ± 0.12	1.49 ± 0.11^∗^	0.95 ± 0.05	1.12 ± 0.07	0.58 ± 0.02	0.70 ± 0.02	0.06 ± 0.01	0.07 ± 0.01

^a^No detection. ∗ and ∗∗ stand for *t*-test under *P* < 0.05 and *P* < 0.01, respectively.

**Table 3:** Correlation analysis between Fe accumulation and leaf chlorophyll content, net photosynthetic rate, stomatal conductance, transpiration rate, and nitrite reductase, succinate dehydrogenase and aconitase activities at stage S3.

Correlation coefficient	Fe concentration	*ISU1* gene expression
Chlorophyll	0.902^∗∗^	0.234
Net photosynthetic rate	0.878^∗^	0.932^∗∗^
Stomatal conductance	0.852^∗^	0.012
Transpiration rate	0.898^∗^	0.334
Nitrite reductase	0. 938^∗∗^	0.874^∗∗^
Succinate dehydrogenase	0.865^∗^	0.915^∗∗^
Aconitase	0.911^∗∗^	0.432

∗ and ∗∗ stand for *t*-test under *P* < 0.05 and *P* < 0.01, respectively.

## Data Availability

All the data used to support the findings in this study have been included in the article.

## Supplementary Materials

Supplementary materials Primer sequences used in this study.
