# Supplementary material for: Foliar Spraying with Compound Amino Acid-Iron Fertilizer Increases Leaf Fresh Weight, Photosynthesis, and Fe-S Cluster Gene Expression in Peach (Prunus persica (L.) Batsch)
Source: Biomed Res Int. 2020 May 25;2020:2854795. doi: 10.1155/2020/2854795 (PMC7273464; doi:10.1155/2020/2854795)
Supplement: Supplementary materials — Primer sequences used in this study. [file 2854795.f1.doc]

Supplementary table 1. Primer sequences used in this study.

| Gene name | Sequence (5’ to 3’) | Amplicon size |
| --- | --- | --- |
| *Ubiquitin* | F: GGCTAAGATCCAAGACAAAGAG AGGCTAAGATCCAAGACAAAGAG | 144 |
|  | R: CCACGAAGACGAAGCACTAAG |  |
| *Actin* | F: GTTATTCTTCATCGGCGTCTTCG  R: CTTCACCATTCCAGTTCCATTGTC | 112 |
| *NFS2* | F: CCTGCCCCCTCAAATCATGT | 190 |
|  | R: AGAAGTGAAGACTCGCACGG |  |
| *SUFE1* | F: TCTCTCAGGTCTGGGTCAGG | 189 |
|  | R: TTAAGCTCTGCTGAAGCCCC |  |
| *SUFE2* | F: TCGTGCCTGATTTGGATGCT | 219 |
|  | R: CGCAAGCAGAGAAGGCAAAG |  |
| *SUFE3* | F: GCAGCAGTTCGTGGTTTGTT | 193 |
|  | R: TAATGGAGCATCCCTCCCCA |  |
| *SUFA* | F: GGTGCTCTGGCATGTCGTAT | 166 |
|  | R: CCTCCACCGATAAGAGCGTC |  |
| *NFU1* | F: GGCACCGGTTAACCTCTCAA | 157 |
|  | R: CGGACGTCCTCAAGAACCAA |  |
| *NFU2* | F: GCCCTTCAGCAACAACCTTC | 184 |
|  | R: CTGCAGTCAGTGACAACTCG |  |
| *NFU3* | F: GGTACGACCTGGCTTGATGG | 152 |
|  | R: TATCTCGCAGGCGGGTCTCA |  |
| *SUFB* | F: AACCCGCCTTGAATAGCCTC | 156 |
|  | R: ACGCCTTCCTATGTGTCGTG |  |
| *SUFD* | F: TTCAGCAACTGGGACCAGAC | 230 |
|  | R: ACTTCTTGTCAGCTGCCCTC |  |
| *HCF101* | F: TCTACAGCCGTGACCTACGA | 236 |
|  | R: CCGTCTGGCCACGTTATTGA |  |
| *GRXS14* | F: CTGTTGGGGCCTTCAACTCA | 170 |
|  | R: GAGCTGAGGTCCAAAAGACGA |  |
| *GRXS16* | F: GTGCCCCATTGTGTGGTTTC | 224 |
|  | R: GGCAAGCTCACCCTTCTCTT |  |
| *NFS1* | F: TCGATTCGTGCCGTCATCTT | 179 |
|  | R: CCCAATTTCCTCCATCGGCT |  |
| *ISD11* | F: CGGAGGTGCTCACACTGTTC | 171 |
|  | R: CGAACTGAGCCTTGCCATCT |  |
| *ISU1* | F: AGACCTTTGGCTGTGGATCG | 247 |
|  | R: GCAGCCTTCTCAACAGGAGT |  |
| *ISA1* | F: CGGCTTGTCGTACACTCTCA | 201 |
|  | R: TCTCCACAACCACACTGTCC |  |
| *ISA2* | F: CTGCCCCTCTCTTCTTCGTC | 161 |
|  | R: GTGCAAGCCCCTCAGTTTTC |  |
| *ISA3* | F: GTCCCTCGATGCTGTTGTGA | 168 |
|  | R: ACTCTGTCATCTGGGTGGGT |  |
| *NFU4* | F: TGCTTATTCACCTGCCACGA | 157 |
|  | R: AAGAGATGCAGGGTTGGGTG |  |
| *ADX1* | F: TTGAAGGAGCGTGTGAAGGT | 161 |
|  | R: CACTTGACAACCCAAGCGAG |  |
| *ADX2* | F: CGTCTGCACCAAAGACAGGA | 246 |
|  | R: CGCAGGATCCTTCACATGCT |  |
| *ADXR* | F: ATCGTTTGCCAACGCCTTTC | 203 |
|  | R: TTCAGCACCATAGGCAAGCA |  |
| *FH* | F: TCAACGATGCTGAAGTCCCC | 173 |
|  | R: GGTCAGCACCTCATTCCCAT |  |
| *HSCA1* | F: CAGCAGCTGACAACCAAACC | 200 |
|  | R: ATCAGTGCCCTTGTCCCTTG |  |
| *HSCA2* | F: AGCCGCAACACAACAATTCC | 154 |
|  | R: GAGGAATGCCCACGAGTTCA |  |
| *HSCA3* | F: ACAGCTGCAGATGGACAGAC | 199 |
|  | R: CTGTGCCCTTATCCACAGCA |  |
| *HSCA4* | F: AATTCCACCAGCTCCAAGGG | 169 |
|  | R: AACCATGCGGTCGATTTCCT |  |
| *HSCA5* | F: AGGATTGTCGCTTGCTTGGA | 215 |
|  | R: AGCCTCCTTCACCATCCTCT |  |
| *HSCB* | F: TCCGGCACTTTCTTCTCACC | 212 |
|  | R: ACAGGTTGAATGCACCGACA |  |
| *INDL* | F: GGTTGGCTTGCTTGATGCTG | 153 |
|  | R: TCCTTGGCCACAAGAAGTCC |  |
| *IBA57* | F: AACGATGTACGGAGGTTCGG | 219 |
|  | R: CTCCAACGCTTCATCTGGGT |  |
| *GRXS15* | F: GAATTTCAGGCTTCCCGGCT | 150 |
|  | R: TAAGGCCAGAACCGTCGAAC |  |
| *ATM3* | F: GCCGGACCTGAGAAATCCAA | 221 |
|  | R: AGCTCAACCAATCCACAGCA |  |
| *ERV1* | F: TCTCATTGGCCAACCTCACC | 233 |
|  | R: CCTTGCAGGGGTACATTCGT |  |
| *NAR1* | F: CGGTGTTGACATCTGGGGAA | 152 |
|  | R: TTCTGCATAACCCCCAGAGC |  |
| *NBP35-1* | F: CCACTCCACAACAGGTCTCC | 152 |
|  | R: ACCGGTCTCTGTCATCCTCA |  |
| *NBP35-2* | F: CCAGAACCTGATGACGCTGT | 210 |
|  | R: GGAGACCTGTTGTGGAGTGG |  |
| *TAH18* | F: CCCACCACCATCACTTCCTC | 201 |
|  | R: AGCACCCCACCATTTTGTGA |  |
| *DRE2* | F: TTGTCCTTTGGGGTCAAGGC | 246 |
|  | R: CTGCTCTTCTTCAGCCCTCC |  |
| *CIA1* | F GAAGCTGGAGGGTCACACAG | 154 |
|  | R: ATCCCACGAAGAAGTGGAGC |  |
| *CIA2* | F: CGCTGTTGAACCCATTGACC | 245 |
|  | R: TGTAACGAGAAGGCAGGCTC |  |
| *CIA3* | F: CCCTTACTCGTTGGAGGAGC | 220 |
|  | R: GGCAGCTTCAGTTGCATGAG |  |
| *MMS19* | F: GCACTGCTGAGGAGGAAAGT | 168 |
|  | R: AATCTCATTCGGATGGCGCT |  |
